# Editorial: Infectious Disease Epidemiology and Transmission Dynamics 2.0

**DOI:** 10.3390/v16081223

**Published:** 2024-07-31

**Authors:** Yuan Bai, Zeynep Ertem, Jose Luis Herrera Diestra, Lin Wang, Zhanwei Du

**Affiliations:** 1 WHO Collaborating Center for Infectious Disease Epidemiology and Control, School of Public Health, LKS Faculty of Medicine, The University of Hong Kong, Hong Kong SAR, China; yybai0523@gmail.com; 2 Laboratory of Data Discovery for Health Limited, Hong Kong Science Park, Hong Kong SAR, China; 3 Systems Science and Industrial Engineering Department, Thomas J. Watson College of Engineering and Applied Science, State University of New York at Binghamton, Binghamton, NY 13902, USA; zeynep@binghamton.edu; 4 Department of Integrative Biology, College of Natural Science, University of Texas at Austin, Austin, TX 78712, USA; diestra@gmail.com; 5 Department of Genetics, University of Cambridge, Cambridge CB2 3EH, UK; fdlwang@gmail.com

This Special Issue includes six original articles and one review article, all reflecting the unified scientific research endeavors and professional expertise for a shared objective, which were published between July 2023 and November 2023. The COVID-19 pandemic unfolded in 2020 [1], and the emergency phase was subsequently resolved in 2023 by layering non-pharmaceutical and pharmaceutical interventions (e.g., vaccines [2] and antivirals [3,4,5]). Following the end of the COVID-19 outbreak, emerging and re-emerging infectious diseases have surfaced or resurfaced, such as Mpox [6], influenza [7], and respiratory syncytial virus [8].

The research includes studies on SARS-CoV-2 variants, such as Alpha, Beta, Delta, and Omicron, focusing on modeling the geospatial spread, analyzing epidemiological indicators, and reviewing the clinical severity of these variants. Vyklyuk et al. [9] improved the previously developed GeoCity model by refining the population’s age structure, incorporating personal schedules for weekdays and weekends, and considering the individual health characteristics of agents. This allowed for a more accurate representation of a city’s functioning. Using this model, they simulated the spread of three SARS-CoV-2 strains (Alpha, Delta, and Omicron) and assessed its applicability in scenarios where the virus spreads freely among city residents. Da Silva et al. [10] analyzed epidemiological data on the incidence, mortality, and case fatality rates of COVID-19 in the state of Paraíba, Brazil, using the Prais–Winsten regression model. Their analysis revealed significant fluctuations in COVID-19 epidemiological indicators from 2020 to 2022, emphasizing the importance of continuously monitoring these indicators as the situation evolves. Yuan et al. [11] conducted a systematic review to evaluate the clinical severity (including hospitalization, ICU, and fatality risks) of various SARS-CoV-2 variants during the period of mass COVID-19 vaccination. Based on 13 studies from nine countries, their findings indicated that the Delta and Omicron variants exhibited the highest and lowest levels of severity, respectively. These insights highlight the need for interventions targeting different SARS-CoV-2 variants and could assist in prioritizing the development of variant-specific vaccines and formulating appropriate treatment strategies.

In addition to SARS-CoV-2 studies, several papers also analyzed other pathogens, including hantavirus, human immunodeficiency virus (HIV), and avian hepatitis E virus (HEV). Cintron et al. [12] developed HantaNet, a hantavirus genomic visualization engine, to monitor and classify viruses, facilitating early detection and response to outbreaks. HantaNet features dashboard visualizations of phylogenetic trees, viral strain cluster classification, epidemiological networks, and spatiotemporal analysis. The authors used 85 hantavirus reference datasets from GenBank to validate HantaNet as a classification and enhanced visualization tool, as well as a public repository for downloading standardized sequence data and metadata to build analytical datasets. Yuan et al. [13] analyzed patient reporting, patient mobility data, and HIV sequence information in Jiangsu Province, China, combining descriptive epidemiology, spatial analysis, and molecular epidemiology methodologies. Their findings indicated a spatial concentration of newly reported HIV infections in Jiangsu Province in 2021, with a reporting rate in southern Jiangsu that exceeded the average. Guan et al. [14] used oropharyngeal and cloacal swab samples from birds collected during routine surveillance of live bird markets in northern Vietnamese provinces between 2018 and 2021 to isolate 77 highly pathogenic avian influenza viruses. They found that these viruses were of the H5N6 subtype and belonged to HA clades 2.3.4.4g and 2.3.4.4h. However, they did not identify any viruses from the 2.3.4.4b clade, which has been prevalent in various parts of the world in recent years. The authors concluded that the highly pathogenic H5 viruses circulating in Vietnam from 2018 to 2021 were distinctly different from those found in other regions of the world, and these Vietnamese H5 viruses continued to evolve through mutations and reassortment. Tsachev et al. [15] investigated the HEV infection positivity rate by collecting 720 serum samples from dogs, cats, horses, cattle, sheep, and goats across seven regions of Bulgaria. They discovered that the overall HEV antibody positivity rate was lower in the Plovdiv and Smolyan regions and higher in the Pazardzhik and Burgas regions.

Finally, we acknowledge all the authors who contributed to this Special Issue. We hope that this Special Issue will inspire more studies on the mathematical modeling of infectious disease epidemiology and transmission dynamics. More research is needed to develop realistic mechanistic models that describe real-world scenarios, as well as improved inferential methods for analyzing actual data.

## References

[1] Du Z., Wang L., Cauchemez S., Xu X., Wang X., Cowling B.J., Meyers L.A. (2020). Risk for Transportation of Coronavirus Disease from Wuhan to Other Cities in China. Emerg. Infect. Dis..

[2] Du Z., Liu C., Bai Y., Wang L., Lim W.W., Lau E.H.Y., Cowling B.J. (2024). Predicting Efficacies of Fractional Doses of Vaccines by Using Neutralizing Antibody Levels: Systematic Review and Meta-Analysis. JMIR Public Health Surveill..

[3] Bai Y., Du Z., Wang L., Lau E.H.Y., Fung I.C.-H., Holme P., Cowling B.J., Galvani A.P., Krug R.M., Meyers L.A. (2024). Public Health Impact of Paxlovid as Treatment for COVID-19, United States. Emerg. Infect. Dis..

[4] Du Z., Wang L., Bai Y., Liu Y., Lau E.H.Y., Galvani A.P., Krug R.M., Cowling B.J., Meyers L.A. (2024). A Retrospective Cohort Study of Paxlovid Efficacy Depending on Treatment Time in Hospitalized COVID-19 Patients. Elife.

[5] Du Z., Nugent C., Galvani A.P., Krug R.M., Meyers L.A. (2020). Modeling Mitigation of Influenza Epidemics by Baloxavir. Nat. Commun..

[6] Du Z., Shao Z., Bai Y., Wang L., Herrera-Diestra J.L., Fox S.J., Ertem Z., Lau E.H.Y., Cowling B.J. (2022). Reproduction Number of Monkeypox in the Early Stage of the 2022 Multi-Country Outbreak. J. Travel Med..

[7] Du Z., Shao Z., Zhang X., Chen R., Chen T., Bai Y., Wang L., Lau E.H.Y., Cowling B.J. (2023). Nowcasting and Forecasting Seasonal Influenza Epidemics—China, 2022–2023. China CDC Wkly..

[8] Du Z., Wang L., Bai Y., Pei Y., Wu P., Cowling B.J. (2023). Mitigation of Respiratory Syncytial Virus Epidemics by RSVpreF Vaccines after the COVID-19 Pandemic in the UK: A Modelling Study. Lancet.

[9] Vyklyuk Y., Nevinskyi D., Chopyak V., Škoda M., Golubovska O., Hazdiuk K. (2023). A Managerial Approach towards Modeling the Different Strains of the COVID-19 Virus Based on the Spatial GeoCity Model. Viruses.

[10] da Silva F.F., de Abreu L.C., Daboin B.E.G., Morais T.C., Cavalcanti M.P.E., Bezerra I.M.P., da Silva C.G., Figueira F.A.M.D.S., de Caldas Guedes V.V., Perez Riera A.R. (2023). Temporal Analysis of COVID-19 Epidemiological Indicators in a Low-Income Brazilian Context: A Retrospective Analysis in Paraiba State. Viruses.

[11] Yuan Z., Shao Z., Ma L., Guo R. (2023). Clinical Severity of SARS-CoV-2 Variants during COVID-19 Vaccination: A Systematic Review and Meta-Analysis. Viruses.

[12] Cintron R., Whitmer S.L.M., Moscoso E., Campbell E.M., Kelly R., Talundzic E., Mobley M., Chiu K.W., Shedroff E., Shankar A. (2023). HantaNet: A New MicrobeTrace Application for Hantavirus Classification, Genomic Surveillance, Epidemiology and Outbreak Investigations. Viruses.

[13] Yuan D., Liu S., Ouyang F., Ai W., Shi L., Liu X., Qiu T., Zhou Y., Wang B. (2023). Prevention and Control Are Not a Regional Matter: A Spatial Correlation and Molecular Linkage Analysis Based on Newly Reported HIV/AIDS Patients in 2021 in Jiangsu, China. Viruses.

[14] Guan L., Babujee L., Browning V.L., Presler R., Pattinson D., Nguyen H.L.K., Hoang V.M.P., Le M.Q., van Bakel H., Neumann G. (2023). Continued Circulation of Highly Pathogenic H5 Influenza Viruses in Vietnamese Live Bird Markets in 2018–2021. Viruses.

[15] Tsachev I., Gospodinova K., Pepovich R., Takova K., Kundurzhiev T., Zahmanova G., Kaneva K., Baymakova M. (2023). First Insight into the Seroepidemiology of Hepatitis E Virus (HEV) in Dogs, Cats, Horses, Cattle, Sheep, and Goats from Bulgaria. Viruses.

